# Scaling and root planing for a patient with perforated palate caused by mucormycosis: A case report

**DOI:** 10.1002/ccr3.7816

**Published:** 2023-08-21

**Authors:** Mohammad Hossein Nikbakht

**Affiliations:** ^1^ Dentistry Student, Student Research committee, School of Dentistry Isfahan University of Medical Sciences Isfahan Iran

**Keywords:** diabetes mellitus, mucormycosis, nasal septal buttons, periodontitis, ultrasonic subgingival debridement

## Abstract

Some diseases like mucormycosis can lead to palatal perforation which can cause limitations for dental and oral care for the patients. Nasal septal buttons may be an effective solution according to the literature.

## Graphical abstract

The case with perforated palate caused by mucormycosis who needed scaling and root planing.

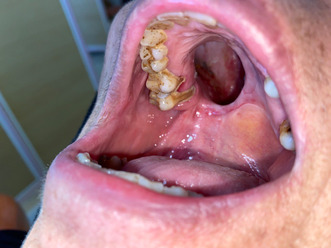

## 1 INTRODUCTION

About 422 million people worldwide have diabetes mellitus.^1^ Polymorphonuclear dysfunction, iron and pH levels, and platelet altering in diabetic patients can be a foundation for rare life‐threatening infection.^2^ One of these diseases is mucormycosis which is a life‐threatening invasive fungal infection. Rhinocerebral mucormycosis (ROCM) is the most common form of mucormycosis in patients with diabetes mellitus.^3^, ^4^ After inhalation of fungal sporangiospores into the paranasal sinuses, the infection develops and may then rapidly extend into adjacent tissues. It can invade the palate, sphenoid sinus, cavernous sinus, orbit, or brain.^5^

Literature confirms that there is a two‐way relationship between diabetes and periodontitis.^6^ Subgingival calculi are covered with a bacterial biofilm that can cause periodontal inflammation. Scaling and root planing (SRP) aimed to remove subgingival calculus and their related biofilm to reduce the inflammatory burden and control the periodontitis.^7^ Hence, SRP can be so beneficial for diabetic patients.

A recent systematic review concluded that there is no significant difference in clinical outcome between hand and sonic/ultrasonic subgingival instrumentation.^8^ However, the ultrasonic instrument is less operator‐dependent and requires less treatment time.^9^

This article aimed to report a case with treated mucormycosis that led to palatal perforation, and my challenges for using the ultrasonic instrument for SRP, because of its need to a water cooling system.

## 2 CASE HISTORY/EXAMINATION

I have read the Helsinki Declaration and have followed the guidelines in this investigation.

A 58 years old woman that lived alone in a rural area attended a clinic for dental scaling. Eight years ago she did not know that she has diabetes mellitus type 2 and her uncontrolled blood sugar led to immune system weakness and mucormycosis infection. The infection has spread to the eye, palate, and mucous membrane on the right side of her face. Surgeons removed infected tissues but the patient went into a coma. After 2 days she regained her consciousness contrary to expectations. However, she could not move her hands and legs. After about 3 months she could move her legs and hands and walk normally. However, her left hand cannot grab and hold things until now.

In the oral examination, she had heavy plaques and calculi all over her teeth, especially in subgingival areas.

## 3 TREATMENT AND OUTCOME

**FIGURE 1:**
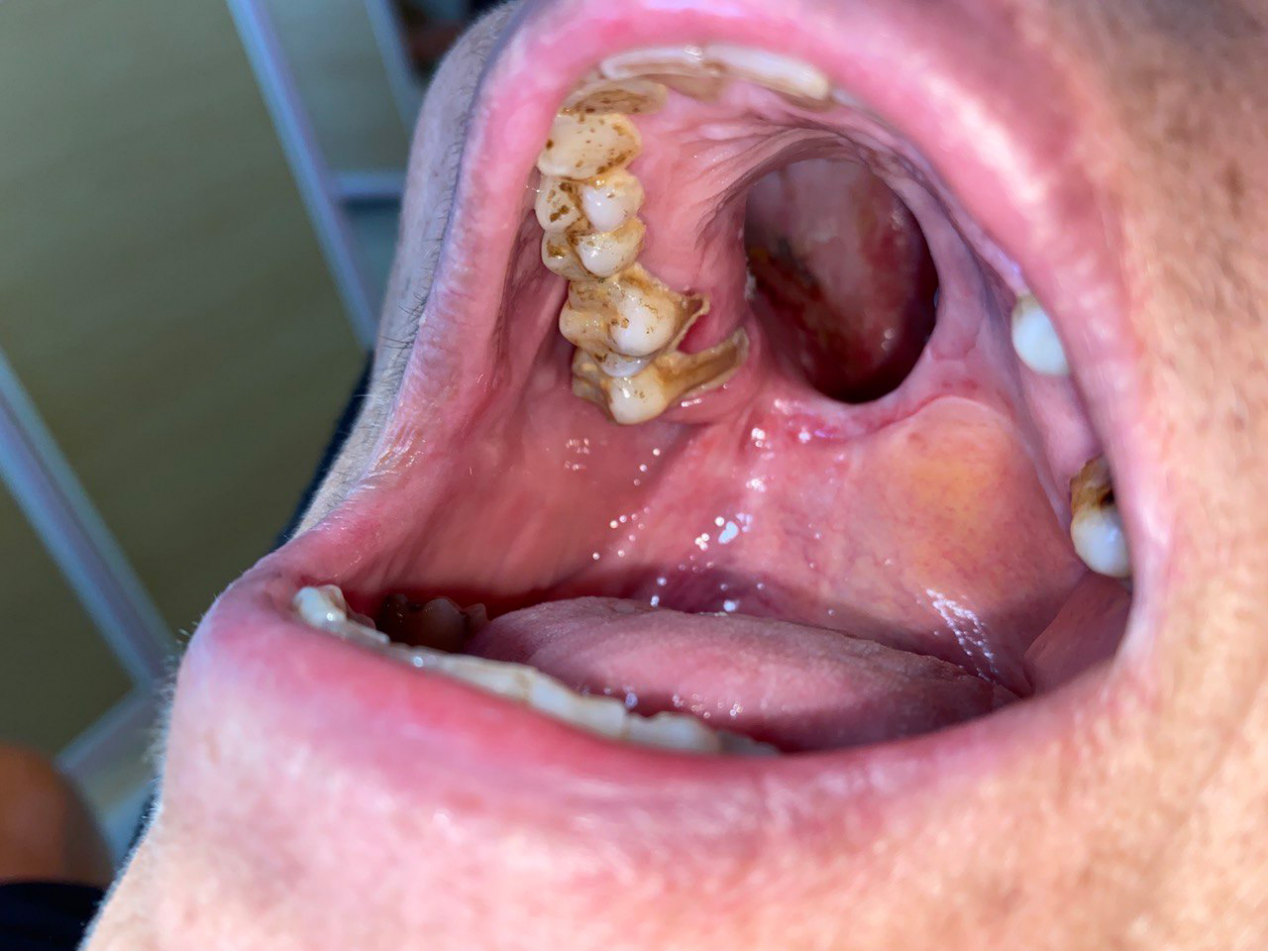
Palatal side of upper teeth before SRP.

Scaling of mandibular teeth and the buccal side of maxillary teeth was performed with the ultrasonic device (Woodpecker—UDS‐K‐LED) in one session. However, the challenge was on the palatal side of maxillary teeth (Shown in Figure 1). As mentioned, she had surgery for the mucormycosis infection and about half of her palate was perforated and exposed to the nasopharynx and orbit. In SRP of the entire mouth except the palatal side of maxillary teeth, which needed to remove the denture, her prosthesis provided the palatal seal. SRP of palatal side of maxillary teeth was performed using conventional curettes.

**FIGURE 2:**
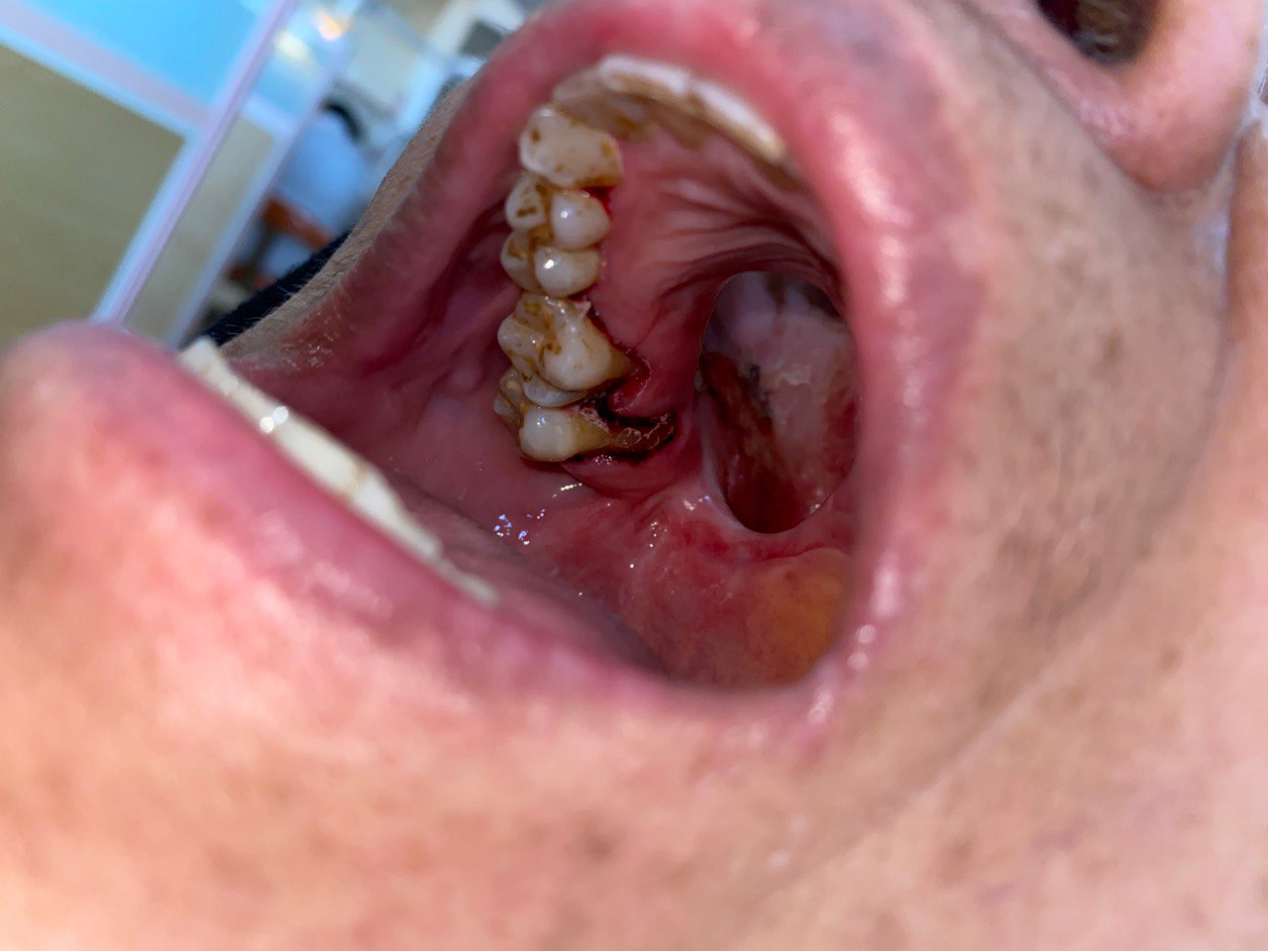
Palatal side of upper teeth immediately after SRP.

SRP was successful immediately after the intervention, no subgingival and supragingival calculus was observed in the clinical examination. Unfortunately, the patient did not come back for the follow‐up. Figure 2 shows the dental condition of the patient immediately after the intervention.

## 4 DISCUSSION

ROCM is the most common form of mucormycosis in patients with diabetes mellitus.^3^, ^4^ And also, the palate is one of the most affected sites in ROCM.^10^, ^11^ Vascular involvement might lead to necrosis of the palate and cause perforation that imposes problems on the patient.^12^

One of the main challenges, in this case, was the limitation of using the ultrasonic device with water cooling to SRP of the palatal surfaces of the teeth that were covered with prostheses. Using conventional curettes could be an option. Although this method is more operator‐dependent and requires more treatment time,^9^ it is enough for an appropriate SRP.^8^ However, using water cooling for other interventions such as tooth restoration is limited for these patients.

A possible suggestion is to make dentures that do not cover the mentioned surface, but usually, these dentures have no appropriate retention. A better solution is to seal the perforation with something that is not extended until the teeth.

Nasal septal buttons (NSB) can be a probable solution to the problem. Different studies examined the efficacy of these buttons for closing perforations (Table 1). Overall, these studies evaluated the use of NSB as successful.^12^, ^13^, ^14^, ^15^, ^16^ Easy application, reduced morbidity, preventing of secondary infections, and prevention of the escape of saliva and nutrients to the nasal cavity are the advantages of NSB. Therefore, it may be an alternative to the temporary repair of hard palate perforation.^12^

**TABLE 1:** Literature review on the studies examined the efficacy of NSB for closing perforations.

First author/year	Usage	Outcome
Khan^13^/1993	Closing pharyngocutaneous fistulas in three cases	Using NSB was an excellent temporizing maneuver in preventing salivary leaks from fistulas
Mirza et al.^14^/2003	Closing a large trachea‐esophageal fistula in a case	NSB was easily inserted, well tolerated, and prevented leakage of solids into the trachea, allowing the patient to eat
Unsal et al.^15^/2015	Closing enlarged trachea‐esophageal punctures for four cases	The NSB closure technique appears to be a simple, safe, and inexpensive method with satisfactory long‐term results
Trimarchi et al.^16^/2016	Repair oronasal communication in a case	They could not follow their patient to determine the long‐term outcomes of this treatment
Kumbul et al.^12^/2021	Closing a mucormycosis‐induced palatal perforation in a case	NSB was well tolerated, and the expected functional results were achieved successfully

I could not use NSB because of its inaccessibility. However, these buttons may solve the problems of using water cooling‐dependent dental tools. It is recommended to design studies to check the efficiency and performance of NSB for this reason.

## AUTHOR CONTRIBUTIONS

**Mohammad Hossein Nikbakht:** Conceptualization; data curation; methodology; project administration; writing – original draft.

## FUNDING INFORMATION

None.

## CONFLICT OF INTEREST STATEMENT

The authors declare that they have no conflict of interest.

## CONSENT

Written informed consent was obtained from the patient and his parents to publish this report in accordance with the journal's patient consent policy.

## Data Availability

The datasets used and/or analyzed during the current study are available from the corresponding author on reasonable request.
